# The Prospective Lynch Syndrome Database: background, design, main results and complete MySQL code

**DOI:** 10.1186/s13053-022-00243-z

**Published:** 2022-11-21

**Authors:** Pål Møller

**Affiliations:** 1 Department of Tumor Biology, Institute of Cancer Research, The Norwegian Radium Hospital, 0379 Oslo, Norway; 2 The European Hereditary Tumour Group, Edinburgh, Scotland, UK

## Abstract

A brief description of why and for which purposes the Prospective Lynch Syndrome Database was established, the principles and design, and the main classes of results are given. Data input is assumption-free input enabling validation of paradigms used to explain the results. The design is considering cancer/age as discrete events to occur or not in a time dimension in a closed room compliant with population genetic paradigms and last centuries developing paradigms of interpreting discrete events reflecting conditional and/or co-occurring stochastic probabilities. Which may be in contrast to the paradigm that any observed event has a cause. The results may indicate that some current paradigms on carcinogenesis should be reconsidered. The complete analytic code in MySQL© syntax together with a flowchart illustrating how the different pieces of codes interrelate are included as supplementary files, enabling third parties to use or modify the code to examine prospectively observed events in their own activities when referring to this report as the source.

**Supplementary Information:**

The online version contains supplementary material available at 10.1186/s13053-022-00243-z.

The reason for making the Prospective Lynch Syndrome Database (PLSD) was that ten years ago it was obvious that the existing paradigms did not completely explain what was being observed: Variants in each of the MMR genes causing Lynch syndrome (LS) had different but uncertain penetrance (cumulative incidence of cancer) and expressivities (organs in which cancer occurred). Because no one has an “average genetic variant” or an ‘average gender’, the “average” penetrance and expressivity were valid for no one. It was known that colonoscopy did not prevent colon cancer as had been hoped (which is why the advocated interval between colonoscopies was reduced in updated clinical guidelines), but the true incidence of colon cancer in those at risk who had regular colonoscopy was not known. The PLSD was designed to describe penetrance and expressivities of cancer by age, gene and gender, survival when cancer occurred and survival in healthy carriers of pathogenic MMR variants when interventions including colonoscopy interfered with the natural history associated with LS.

Empirical evidence is needed from prospective studies to obtain more accurate information on prospective incidences of phenotypes. Three examples why retrospective information may not predict future risks for inherited phenotypes may be: (1) In a stable population, 33% of woman will have a sister when complete selection ascertainment, while the probability for an expecting woman that her baby will be a girl is about 50% irrespective of any previous girls she may have delivered. (2) With multigenic inheritance, the next generation will have a reduced phenotypic incidence. (3) Retrospective ascertainment biases may cause an artificial increase in phenotypic incidence (anticipation).

When validating paradigms, no information entered into the database should be restricted, ascertained, conditioned or based upon the paradigms that are to be validated (you cannot have your ascertainment parameter as your study parameter). The design of the study should not be restricted to the examination of one specific paradigm or claim – if restricting your design to examine one hypothesis, the results will be nothing but a validation of that specific hypothesis. In the context of LS, if two or more carcinogenetic pathways may be operating simultaneously, proving an adenoma to be a precursor of cancer does not exclude other carcinogenetic pathways to colon cancer. The idea of the PLSD was to measure by how much colonoscopy reduced colorectal cancer incidence – it came as a surprise that colorectal cancer was apparently not reduced as a result of colonoscopy [1]. The design of the PLSD, however, did not include any initial paradigm and the design allowed unexpected results: The PLSD reports on the observed empirical evidence based on assumption-free data. This does not, however, exclude ascertainment biases which any study will have and in relation to which results must be appropriately interpreted [2].

Most studies of carcinogenesis and cancer clinical trials explore or test one or a few hypotheses, the ‘gold standard in oncological research’ of a randomized trial being the classical example. In doing so, you will find nothing besides what you are looking for: you may describe one tree in detail, but you may not see the forest.

PLSD was designed as follows: The primary object (never to be duplicated and never to be split into two) is the carrier, and to the carriers are attached the attributes gender and genetic variant which are inborn and will never change. The events recorded are any cancer/age when diagnosed, age at inclusion for follow-up, age at last observation, and age at death. The analytic model needs an adequate number of carriers included in all age groups, and especially so in the youngest age groups when calculating cumulative incidences. Power analyses indicated that about 2,000 carriers should be included for a first analysis that did not stratify on gender [3]. As, at the time, no single centre or even country had enough carriers to provide the required granularity in their data, in 2012 members of the Mallorca Group (now EHTG www.ehtg.org) agreed to compile data on their prospectively observed carriers. The increased number of cases later included allowed more detailed analyses.

To handle such numbers of cases, a computerized database is required: The basic principles of the PLSD relational database and how it was de-normalized for the current task have been previously described [4, 5]. Also, the major confounders to be considered when interpreting the results in oncological research like this have been previously discussed [2]. The basic PLSD administrative description and data call is available online https://www.ehtg.org/plsd.php. While the PLSD reports present cumulative risk for cancers by gene and gender from 25 years of age onwards, cumulative risks starting from any current age of 25 years or more may be calculated interactively at www.plsd.eu, based on the last PLSD report. This may be of interest to carriers of different ages.

The PLSD reports so far indicate that each of the four LS genes when deranged are associated with different inherited cancer syndromes. All cancers included in the LS spectrum (except for brain tumours and osteosarcomas) are derived from the embryonic endothelial tissues, but occur with different incidences in the different affected organs according to the respective germline pathogenic variant.

- The MSH2 syndrome is a truly multi-organ dominantly inherited cancer syndrome frequently having extra-intestinal cancers in the urinary tract, prostate and brain and in women frequent endometrial and ovarian cancer. Colonoscopy may be associated with over-diagnosis of colorectal cancer.
- The MLH1 syndrome has high incidences of gynecological, colorectal and upper intestinal cancers with a higher incidence of colorectal cancer in males than in females. Colonoscopy may be associated with over-diagnosis of colorectal cancer.
- The MSH6 syndrome is by and large a dominantly inherited sex-limited cancer syndrome having a high incidence for endometrial or ovarian cancer and a slightly increased and gender-equal incidence of colorectal cancer. It is under-reported because males often appear as skipped generations in families. There is no evidence that colonoscopy reduces incidence of colorectal cancer.
- The PMS2 syndrome has lower but increased incidence of endometrial and colorectal cancer but seemingly no validated increased risk for cancer in other organs. It is under-reported because low penetrance makes it difficult to delineate from normal variation. In contrast to the others, colonoscopy may reduce the incidence of colorectal cancer before 50 years of age.

Ten years survival following early diagnosis and treatment of colorectal or endometrial cancers in carriers of pathogenic variants of *MSH2* or *MLH1* is high. For carriers of pathogenic variants of all four genes who are subjected to follow-up as advocated by international guidelines, death from extra-colorectal cancers now seems more frequent than from colorectal cancers, especially so for carriers of pathogenic variants of *MSH2* and *MSH6. MLH1* carriers may die from later pancreatic or biliary tract cancers. Risk from dying from ovarian cancer diagnosed before 40 years of age for carriers of any genetic variant was calculated to 0%, concluding that prophylactic oophorectomy before that age may not be indicated [6].

Making the PLSD MySQL algorithms available to all should facilitate others in validating the published PLSD results, which is a general requirement for trusting new information that is used for health care. For the reason the algorithms are here made available for all in the supplementary files: The complete MySQL codes for all tables, views and functions; a flow-chart on how the different parts interact; installation notes; and a few cases to populate the tables as a demonstration for a start. It runs on the free version of MySQL8©. Tables may be populated by importing the data from any source having an ODBC driver; any of the final outputs will be displayed within a second or two upon request and results may be exported for further processing to any other application having an ODBC driver. The MySQL outputs are in three classes: (1) Number of events, number follow-up years and the fraction as annual incidence rates in each age cohort for any filtering selection as specified; (2) output tailored for K-M survival analyses when certain events are under consideration; and (3) some counts of cases included by carrier status and geographical region lived in. Additional tables including more attributes of the carriers may be added to the MySQL database, and additional views/functions may be added to answer additional questions and to analyze more substrata. Further calculations of cumulative incidence should be based on Poisson distributions as previously published [1].

The design of the PLSD database (cancers considered as discrete events by age) is compliant with the requirements included in population genetic theories, including closed room statistics like hypergeometric distributions, Boolean parameters and Bayesian probability calculations, etc. when appropriate. The design is also compliant with the new paradigms on stochastic probabilities as causes for events, which may be considered in contrast to Newton’s paradigm of any event having a specific cause, and this may be of interest for understanding carcinogenetic mechanisms.

## Supplementary Information

**Additional file 1.**

## Data Availability

All included in supplementary files.
