# Supplementary material for: The Prospective Lynch Syndrome Database: background, design, main results and complete MySQL code
Source: Hered Cancer Clin Pract. 2022 Nov 21;20:37. doi: 10.1186/s13053-022-00243-z (PMC9677689; doi:10.1186/s13053-022-00243-z)
Supplement: Supplementary file 1 — Additional file 1. [file 13053_2022_243_MOESM1_ESM.zip › PLSD_MySQL_FLOWCHART_HCCP_221019.pdf]

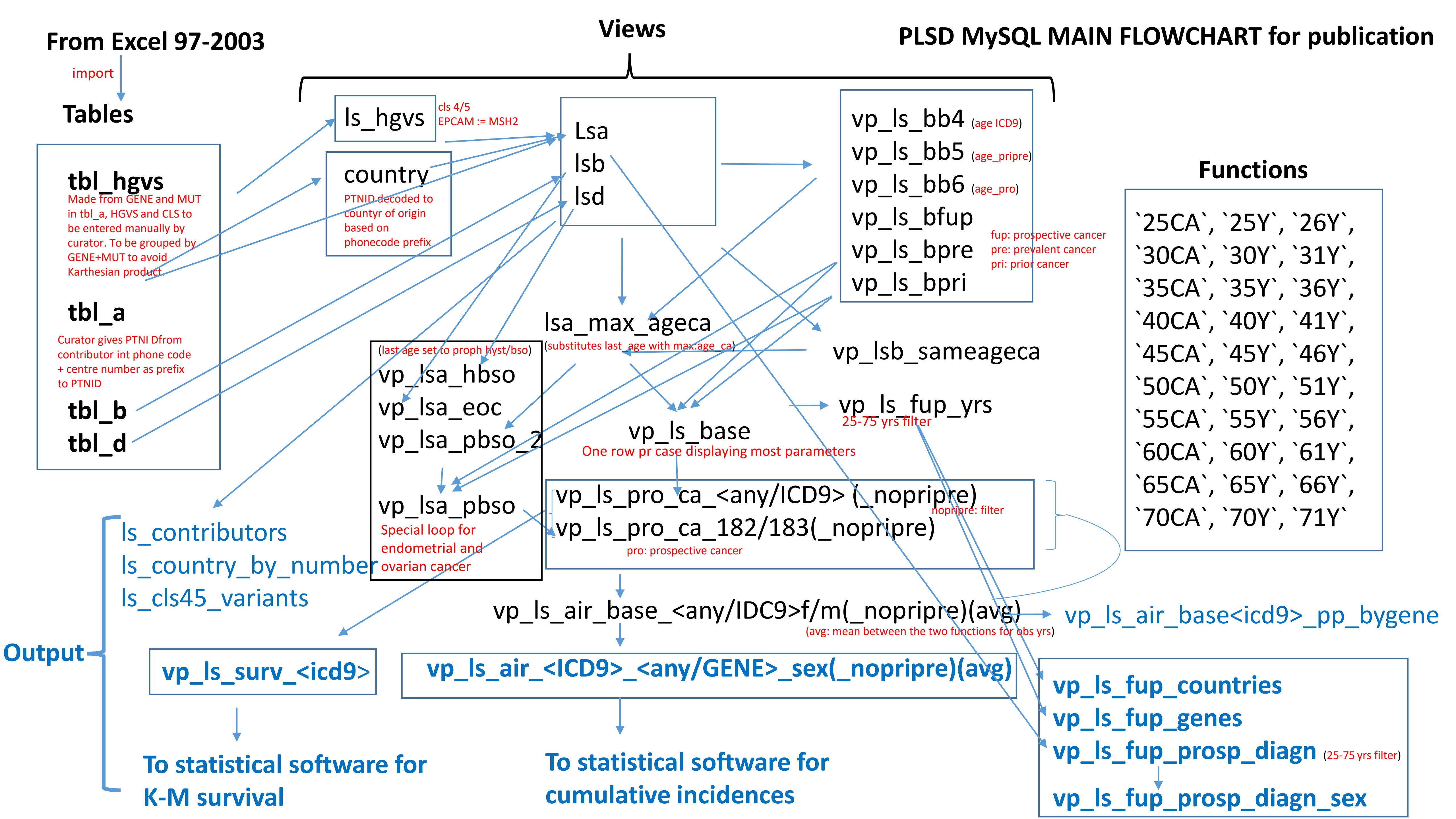
PLSD MySQL MAIN FLOWCHART for publication
From Excel 97-2003
import
Tables
Views
Functions
tbl_hgvs
Made from GENE and MUT in tbl_a, HGVS and CLS to be entered manually by curator. To be grouped by GENE+MUT to avoid Karthesian product
tbl_a
Curator gives PTNI Dfrom contributor int phone code + centre number as prefix to PTNID
tbl_b
tbl_d
ls_hgvs
cls 4/5
EPCAM := MSH2
country
PTNID decoded to countyr of origin based on phonecode prefix
Lsa
lsb
lsd
vp_ls_bb4 (age ICD9)
vp_ls_bb5 (age_pripre)
vp_ls_bb6 (age_pro)
vp_ls_bfup
vp_ls_bpre
vp_ls_bpri
fup: prospective cancer
pre: prevalent cancer
pri: prior cancer
`25CA`, `25Y`, `26Y`,
`30CA`, `30Y`, `31Y`,
`35CA`, `35Y`, `36Y`,
`40CA`, `40Y`, `41Y`,
`45CA`, `45Y`, `46Y`,
`50CA`, `50Y`, `51Y`,
`55CA`, `55Y`, `56Y`,
`60CA`, `60Y`, `61Y`,
`65CA`, `65Y`, `66Y`,
`70CA`, `70Y`, `71Y`
lsa_max_ageca
(substitutes last_age with max:age_ca)
vp_lsb_sameageca
(last age set to proph hyst/bso)
vp_lsa_hbso
vp_lsa_eoc
vp_lsa_pbso_2
vp_lsa_pbso
Special loop for endometrial and ovarian cancer
vp_ls_fup_yrs
25-75 yrs filter
vp_ls_base
One row pr case displaying most parameters
vp_ls_pro_ca_<any/ICD9> (_nopripre)
vp_ls_pro_ca_182/183(_nopripre)
nopripre: filter
pro: prospective cancer
ls_contributors
ls_country_by_number
ls_cls45_variants
Output
vp_ls_air_base_<any/IDC9>f/m(_nopripre)(avg)
(avg: mean between the two functions for obs yrs)
vp_ls_air_base<icd9>_pp_bygene
vp_ls_surv_<icd9>
vp_ls_air_<ICD9>_<any/GENE>_sex(_nopripre)(avg)
vp_ls_fup_countries
vp_ls_fup_genes
vp_ls_fup_prosp_diagn (25-75 yrs filter)
vp_ls_fup_prosp_diagn_sex
To statistical software for K-M survival
To statistical software for cumulative incidences
