# Supplementary material for: The Prospective Lynch Syndrome Database: background, design, main results and complete MySQL code
Source: Hered Cancer Clin Pract. 2022 Nov 21;20:37. doi: 10.1186/s13053-022-00243-z (PMC9677689; doi:10.1186/s13053-022-00243-z)
Supplement: Supplementary file 1 — Additional file 1. [file 13053_2022_243_MOESM1_ESM.zip › Installation notes.doc]

Initially create the schema/database PLSD in your MySQL.

The script file includes creation scripts for each table, view and function which may be copied into the edit panel and run. The code was generated by TOAD for MySQL© v7.5.0.966 from local instance of MySQL© v8.0 running on a Surface_Pro_3©i7 and stored in Wordpad©. First create the tables. A database does not like empty tables and an Excel© workbook with three cases to populate the tables is included and may be imported for a start if you like to (these are to be deleted when later populating the tables with your data). Then run the creation scripts for the views and functions over and over again until error message is that they all already exist. When completed, there should be 4 tables, 265 views and 30 functions. Until sufficient data in the tables to populate all views are entered, some views will be empty. One way of understanding how it works when implemented, is to start with the tables and launch each table and view as they come in the flowchart to see the return value as tables in the MySQL grid, and then read the code for each view/function to see exactly how the return values are made. The multiple views facilitate debugging, maintenance, development, simplifies the process if wanting to migrate the code to another database, reduces demand to computer capacity and increases processing speed.

Tbl a, b and d are populated by importing your data. Tbl a includes constant characteristics, PTNID is the primary key. PTNID is a VARCHAR string given by contributor, to with the curator add the international phone-code for residence + the contributors number in the country decided by the contributor as prefix. This will secure no PTNIDs to be identical, and facilitate categorizing cases by country and contributor. There is no constraint besides PTNID being the primary key in tbl a, the tables are to be manually curated for referential integrity. Tbl b indicates cancers/ages. Tbl d indicates prophylactic oophorectomies and hysterectomies (organ removed coded as ICD9 code R_ICD9 when cancer in organ).

Tbl_HGVS is populated by copying column GENE and MUT from tbl a while grouping them by GENE+MUT and populating column CNT as numbers in group (grouping needed to avoid downstream Karthesian product if more than one row with same GENE+MUT). Correct HGVS nomenclature and pathogenic class (CLS)copied from <http://insight-database.org/> is manually given by curator of the database. GENE+MUT is relational key for including the two curated parameters into lsa. Lsa includes cls4/5 variants from tbl_a where EPCAM is converted to MSH2. Lsb is an inner join between tbl b and lsa, lsd is an inner join between tbl d and lsa. Lsa, lsb and lsa are in effect the ‘tables’ in the relational database with lsa as parent table and all relations are based on PTNID. User may analyze subgroups by modifying the script for the lsa view.

Two techincal statistical notes related to filing age as an integer: There are two ways of calculating observation years, the functions do both ways, to a view which sums them and divides the sum by two. The K-M survival algorithm will exclude a cancer case having died at the same age as diagnosed, which is mended by giving such cases 0.5 years survival.

Because information on oophorectomy when hysterectomy, vice versa, and similar for ovarian and endometrial cancer were not to be trusted, there is a separate loop right-censoring observation years for the one when the other.

When correctly installed, the view vp_ls_base will have the same row count as the view lsa, having the arguments for each case in one row per case.

The free version of TablePlus© is suitable for backing up and restoring the complete database.
